# The British Hospitals Association and the National Insurance Bill

**Published:** 1911-12-09

**Authors:** 


### THE BRITISH HOSPITALS ASSOCIATION AND THE NATIONAL INSURANCE BILL.

### A Bill Injurious to the Voluntary Hospitals and Grievously Cruel to British Workmen.

A largely-attended conference of the British Hospitals Association, to consider the National Insurance Bill as amended, was held at the Westminster Palace Hotel on Friday afternoon, December 1. The Chair was taken by Mr. A. William West, Chairman of the Council, and among those on the platform were the Right Hon. Alfred Lyttelton, M.P., Canon Hensley Henson, Mr. C. S. Loch, Sir Henry Craik, M.P., Mr. James Boyton, M.P., and members of the Council. Representatives of nearly two hundred hospitals situated in all parts of the country were present.

Dr. D. J. Mackintosh, the honorary secretary, read the names of those who had sent apologies for absence, including that of the Hon. Harry Lawson, M.P., who wrote that he was looking after hospital interests elsewhere; and also gave the substance of communications from various centres where meetings had been held and resolutions passed, substantially similar to those before the meeting that day.

The Chairman said that the meeting was called by the British Hospitals Association, consequent upon the very widespread desire of hospital administrators to consider the position of voluntary hospitals under the Insurance Bill. As at present framed the Bill meant an end of voluntary institutions. This was the general opinion of all hospital men with whom he had had to do. Subscriptions, donations and legacies must inevitably fall away. Already notices of withdrawal of subscriptions had been forthcoming; and here he read two typical letters, one from an employer who from inquiring among his men had found that in the majority of cases they would cease their subscriptions when required to pay the Government tax, and the other from a personal subscriber, an employer in a small way, who regretted having to discontinue his subscription, which in this case was a couple of guineas. Yet the Bill could hardly fulfil its purpose unless institutional treatment were given, and obviously this could not be done if the funds were depleted. Again, what about medical education and the training of nurses? Any reduction in the number of beds in the hospital, and in its efficiency, meant also a reduction in the number of men who could be trained. It would be a disaster if the hospitals were no longer able to take in all cases which needed institutional treatment. If the hospital beds were reduced an infinity of harm would be done. He trusted that by some means they would be able to continue the voluntary system. There were many other aspects of this question into which he did not propose to go, but he thought it was a matter of regret to everyone in that room that the Bill should have been rushed through. (Hear, hear.) He concluded by calling upon Mr. Alfred Lyttelton to move the double resolution which stood in his name.

The Right Hon. Alfred Lyttelton, K.C., M.P., moved two resolutions, which were subsequently combined as follows :—

"That this Meeting records its conviction that hospital treatment as at present provided cannot be adequately maintained by the voluntary hospitals if the existing sources of income are seriously diminished, as in the opinion of this Meeting they are likely to be in consequence of the provisions of the Bill; and in such case the British Hospitals Association again affirms its unanimous opinion that the National Insurance Bill is incomplete unless it provides for the hospital treatment which insured persons must have if their medical needs are to be covered by the Bill."

### THE RIGHT HON. ALFRED LYTTELTON, K.C., M.P.

The audience I am addressing will fully agree, I think, with the statement that the voluntary system has brought with it many and signal benefits, and will feel that the splendid tradition which has been built up in 150 years should not, at any rate without a gallant fight, be dispersed. I suppose that I have been asked to move this resolution, although there are so many gentlemen who are far greater experts than I in this company, because I am a member of Parliament, and therefore deemed—I say advisedly, deemed—to be acquainted with the provisions of the Insurance Bill. He would be a bold man who, even though he had given an enormous amount of study to the Bill, could say with assurance that he thoroughly grasped its provisions. If there is a gentleman present who can make that claim, I hope that he will be merciful to me. (Laughter.)

This Bill, so far as I have been able to gather from speakers who have advocated it in the House, has one principle which is fundamentally sound. It seeks to deal with disease at the earliest possible stage and to deal with it effectively. But obviously such provisions as the Bill makes in this respect must be supplemented for a very long time to come, particularly so far as concerns serious cases of illness and disease and operations of anything like a complicated character. If the scheme of insurance is to be called with any colour of pretence a national scheme, it must not cease with the domiciliary visits of the general practitioner. An organisation of a high and efficient kind must exist for the purpose of dealing with disease in its more serious categories, with operations, with chronic heart complaints, with cancer, and other conditions of that kind. When I say "serious" I mean disease which carries with it the necessity of treatment—and prolonged treatment—under better conditions than the poor can possibly secure in their own homes. Hospitals therefore—I do not think anybody denies it—must continue. Their facilities for training medical men and nurses, their great organisation for research work, their statistical records and observations in pathology, must remain. And here I come upon ground where there is not such universal agreement. It seems to me that the opportunity which will be brought before the hospital world by the passage of the Insurance Bill will be for the closer definition of the functions of the hospital, and also perhaps the closer delimitation of those functions. (Hear, hear.) I got into great trouble with Mr. Sydney Holland for saying this; nevertheless, I imagine that when we have an organisation such as is proposed in this Bill, it would be a waste and overlapping for the hospitals to continue in the same degree as hitherto their great system of out-patients. (Hear, hear.) I think it absurd that after employer, employé, and the State have joined to contribute the 9d. per week, the insured person should have the right to go to the hospital and say, "Cure me of this or that." Mr. Sydney Holland said to me that it would be out of the question to get the young doctors trained unless the lighter class of cases were taken as well as the heavier. But surely arrangements might be made with the dispensaries for that comparatively minor purpose?

But before there is any violent alteration of the hospital system there must, as the Chairman has pointed out, be inevitable loss. It is obvious that men who pay, as some employers will do, thousands a year for their employés in respect of the National Insurance Bill, cannot be expected to keep up their subscriptions to the voluntary hospitals. And equally true is it with regard to the smaller subscriptions of the employé. We tried to influence the Government to see that point of view and to provide that some portion of the benefit should be paid to the hospital when the hospital did the service. The clause of the Bill which deals with that question seems to give rather faint hope of much subsidy. It places three conditions as precedent before any such arrangement is made. Taking the case of a man who is brought into the hospital and receives very substantial benefit, no contribution can be made to the hospital for that man unless three conditions are fulfilled : (1) that there is an agreement between the hospital and the approved society; (2) that he has no dependents; (3) that he belongs to an approved society. Two of these conditions are very serious. The humblest class of all will not belong to an approved society, and will still receive the benefits the hospital has performed. Further, there will be a wonderfully fertile crop of dependents. (Laughter.) I do not think that the proposition that there might be contribution requires an agreement. It seems to me so obviously wrong that people should receive from the hospital those benefits which are virtually in relief of the insurance scheme and the hospital get nothing for it. Finally, I do not think we need be discouraged because of the imperfect success we have had with Mr. Lloyd George. Hopes are always to be entertained of the Chancellor of the Exchequer. He has shown a capacity for rapidity of conversion for which we must go to religion to find a parallel. (Laughter.) And the case for the hospitals rests on such solid argument that I do not think we need be downcast because of preliminary failure to make an impression. (Cheers).

### CANON HENSLEY HENSON

in seconding the resolution, said he had been unable to find anyone who, having earned the right to have an opinion on social questions, was not tremendously exercised in mind as to the consequences of this hasty and continually changing measure. Those who were best entitled to do so offered criticism of such cogency and such importance that no reasonable man could avoid the gravest anxiety as to its ultimate results. As a citizen, separated by his position from all contact with party politics, and interested by reason of his profession in all matters concerning the lives of the poor, he protested against the indecent haste with which this immense measure had been carried through. That afternoon they had met to consider only one, but not the least important, of its indirect consequences. It would reduce the income and thereby lower the efficiency of our hospitals which, as they now existed, were the best hospitals in the world. It was morally certain that it would mean sooner or later the destruction of their voluntary character. He did not often find himself in agreement with Mr. Philip Snowden, but he endorsed the words of the Labour leader when he wished for a form of public opinion strong enough to induce the withdrawal of the Bill so that after fuller consideration a better measure might be introduced. The Insurance Bill affected the hospitals in various ways. It increased their expenditure. To insure all the employés of the London Hospital would cost something over £800 per annum. It would decrease their income from voluntary contributions. Two-thirds of the income of the London Hospital was derived from such contributions, and if he mentioned the London Hospital it was owing to his past experience of that institution when he was its neighbour and a constant visitor. It was impossible not to believe that when a compulsory burden was laid on employers and employed their contributions would cease. Again, the contributions to the hospitals were made, not only by employers and employés, but by benevolent people with small fixed incomes who, with additional charges laid upon them in respect of taxation, might find it impossible to give their customary donation. Legacies, again, were a very important factor, and were likely to be influenced by the same causes that would bring about a decline in voluntary contributions. If this Bill passed he felt that their fears would be justified in the event, and the number of beds must be reduced and their whole efficiency lowered. At present hospitals existed in the large liberty of a voluntary system. They co-operated and fitted in with one another. The Bill would reduce them to a dead level. They kindled local enthusiasm and enthusiasm among their doctors, their nurses, their managers, even their patients. This could scarcely survive State management, with which they were threatened. The speaker read the report of an officer of health as to the results of medical inspection of schools. It stated that the service of voluntary agencies was superior in many ways to that of public authority. The voluntary worker contributed initiative and invention, he lavished exceptional care upon difficult problems, he was at liberty to experiment, he often brought to bear upon the work a religious spirit and motive, which, of course, were not necessarily absent in the case of a paid official, but were likely to be less in evidence. This was the direction, he regretted to say, of national policy in the matter of social work. Formerly the State encouraged voluntary agencies, entering indirectly as an ally and an adjunct. Now the voluntary agencies were being directly replaced, and every kind of social work was being undertaken at the public cost. It was threatening to destroy the fund—not an infinite fund—of voluntary bounty and to dishearten voluntary effort. The agreement between the approved society and the hospital, to which Mr. Lyttelton had referred, was really the thin end of the wedge of public control. It would only be a matter of time before hospitals came on the rates and taxes. There was no mention of control, truly, in Clause 20 which dealt with the matter, but it was morally certain that if the clause were acted on in any considerable degree a demand for control would sooner or later be made. (Cheers.)

### SIR HENRY BURDETT, K.C.B., pointed out that never before in the history of this country had there been held so representative and important a meeting to consider provision for times of sickness as on that occasion. They had representatives from two hundred hospitals scattered all over the United Kingdom, and for the first time London and the provinces, Scotland and Wales, were all united in a single effort. The Bill was not business. It took away from the working-classes the hospital provision which they at present enjoyed through the voluntary institutions, and it put nothing in its place. He ventured to say also that their whole difficulty that day was due simply to the fact that this most momentous and vast change in our social system was being so hurriedly attempted, while its provisions were so intricate that probably not even Mr. Lloyd George himself could pass a preliminary examination in it. (Laughter.) The speaker was one who was acquainted with every class of hospital, and with hospitals all over the world, and he was bound to say that their own system of hospitals was recognised to be the very best that the world had yet produced. When the Bill was passed half the hospital provision which the country enjoyed from the voluntary institutions would be taken away. The Bill tapped the sources from which their resources came, and the tapping could not do less than reduce the contributions of the voluntary hospitals in London by 23 per cent., in the provinces by 48 per cent., in Scotland by 44 per cent., and in Ireland by 29 per cent. That reduction would be materially increased when regard was taken of extraordinary income, which included legacies. London hospitals obtained at present 25 per cent. of their income from legacies, provincial hospitals 32 per cent., Scottish hospitals 52 per cent., and Irish 7½ per cent. These figures were all available and open, from audited statements of accounts, and anyone who questioned a single item might come to him (Sir Henry) and he would prove them up to the hilt.

The best definition of the position after the passing of the Bill might be given in Mr. Lloyd George's own words. On July 29 a deputation from the British Hospitals Association met Mr. Lloyd George in order to discuss the question, and in his reply, speaking of the loss of income to the voluntary hospitals, the Chancellor said that the remedy for this loss of income was very largely in their own hands. The hospital should ask the patients after the passing of the Bill, "Are you insured?" If they were, they had no right to come to the voluntary hospital Unfortunately, the whole speech was not reported in the daily Press, but a verbatim report was taken by the *Times* reporter. This appeared in The Hospital, a copy of it was sent to Mr. Lloyd George, and the statement remains uncontradicted. It came to this, then, that it was not only a case of injury to the hospitals, but a case of the working-men throughout the country being affected by the Bill. The bread-winners could not maintain themselves unless they had hospital provision, but according to Mr. Lloyd George, after their own payments, their employers' payments and the State contribution, they became ineligible for the voluntary hospitals. Where, then, were they to go? There was no other hospital accommodation for them whatever. Owing to the increasing efficiency of the voluntary hospitals during the last twenty-five years, the average number of days' residence of the working-man patient had been reduced from 35 to 18 or 19 days, and that represented in London alone an addition each year to the earning power of the working-classes of one million working days. That was a striking fact—(hear, hear)—and it proved that the working-men must have and could not do without hospital treatment. A measure involving such ineligibility would be a cruel measure. (Applause.)

Again, to quote Mr. Lloyd George, on introducing the Bill the Chancellor was reported to have said that it provided free medical relief with no taint of charity. If there was to be no taint of charity, it was quite clear that the insured could not continue to enjoy the hospital benefit that they did enjoy under existing arrangements. At Birmingham on June 11 the Chancellor said: "The first thing we do in our Bill is to provide adequate medical treatment for every workman in the United Kingdom." This claim was confuted by the Bill itself. The workman became ineligible; the doors of the hospital must be closed to him. And if they said, as benevolent people, that the poor workman must have hospital care as before, the hospitals having lost half their revenue could only maintain half their present number of beds, and certainly every single bed they could retain would be required by the wives and children of the insured and by the sick poor whom it was their special pride and function to relieve. Clause 14, upon which the last two speakers had touched, was not worth considering financially at all. It did not bear financially any closer relation to the cost of the in-patients by the provision it was likely to make, than their public alms-boxes bore to the whole of their average income. Nor could they depend on Clause 20, which made it lawful for the approved society or local health committee to grant such subscriptions and donations as they might think fit. If these societies gave charitable contributions, that money must in justice first be devoted not to the insured applicants, but to their wives and children. That did not touch hospital provision. Sir Henry considered that the withdrawal under this Bill of hospital care would bring an ocean of new and avoidable miseries to the workmen all over the country. In all probability it would vitally affect, not only the voluntary foundations of the hospitals, but most cruelly the homes of the working-classes, too, as they exist to-day.

There was an alternative, which, he regretted to find, some people after insufficient thought about the matter were supporting. This alternative was to wipe out voluntary hospitals and have State hospitals. But the Poor Law Commission, when they went into this question, estimated that the cost of State hospitals in this country could not be less than twenty millions a year. That estimate had since been confirmed. He (the speaker) went fully into the question with the authorities who had most to do with these matters, and found that £20,000,000 was the very lowest sum that could be suggested. If, therefore, this Bill in addition to its cost in other directions were to abolish voluntary hospitals, it would mean a total addition to the national expenditure of forty millions a year. Where was the money to come from? There was one other point. If they had State hospitals, what would be the effect upon the public? We owed to our present system of hospitals our medical care, our nursing service, the development of various kinds of treatment, and an influence in the direction of hygiene and health-building which extended far into the homes of the people. But in State hospitals, according to those who had thought most deeply on the subject, all this would be different. The patient was the first consideration in the voluntary hospital, but under the State system the patient was liable to become increasingly an object of scientific interest, and his individual interests were apt to pass further and further away. He thought the sooner they had an end to this hurried, rash, and ill-considered pressing forward of so-called legislation, however meritorious the motives which led to its pursuance, the better it would be for the safety and welfare of all classes throughout the country. (Applause.)

### SIR HENRY CRAIK, K.C.B., M.P., in supporting the resolutions said that, as the representative of five thousand members of the medical profession, he had honestly tried to follow the intricacies of the Bill, especially on its medical side. It seemed to him that at present there were three classes in the country to which the hospital system naturally adjusted itself. There were the very poor, whom they had always with them. There were those who formed the membership of the Friendly Societies. And beyond these there were the very large number of well-to-do working men earning from thirty shillings to three pounds per week, who as a rule chose their own doctor, formed his friendship, and remunerated him fairly. The voluntary hospitals opened their doors to the very poor, and, on occasion and when necessary, to the other classes, particularly when the men could not pay for operations or for a long course of treatment. Under the Bill they were now to be brought down to one low level. The people who had their expectations aroused by the Bill, and considered themselves insured for all necessary medical treatment would certainly be prepared to claim the right to medical treatment in the hospitals, with the result that the hospitals would be inundated with cases at the very time that the sources of benevolence and voluntary aid would be cut away. The proper remedy would be to restrict the medical aid part of the Bill to those who absolutely required it, to the man earning thirty shillings or no more than £2 per week. He believed that if Mr. Lloyd George had listened to that suggestion the hospital question would have settled itself. The better-class working man would have continued to contribute to the hospital, knowing that occasionally he might have to ask admission. The employer would have contributed, knowing that the hospitals helped those who had tried to help themselves, and the voluntary contributor would have continued to do his part, knowing that the hospitals were upheld by voluntary aid. But, with the levelling down which would take place under the Bill, the whole of the voluntary agencies would be laid under a heavy press, and one of the finest influences would be carried out of our English life. (Applause.)

### COLONEL ROXBURGH (WESTERN INFIRMARY, GLASGOW), after pointing out the many-sidedness of the criticism which had been directed upon the Insurance Bill, said that the criticism of the managers and administrators of the hospitals differed from the others in the respect that, personally, *they* had nothing to gain or lose by the Bill becoming law. Their strictures were purely disinterested, and arose out of the conviction that the provision for those insured under the Bill in case of severe sickness was not adequate, and that by reason of this circumstance the facilities at present existing would almost certainly be killed. Hospital managers were not against the principle of the Bill. Perhaps more than others, they realised the desirability of some such provision as it attempted to make, but they were not blind to its inconsistencies. They felt convinced, further, that if the Bill passed in its present state it would materially prejudice hospital work, while it made no provision for superseding it. They believed in the voluntary principle which had made the British hospitals what they were, and which served the patient better than would be possible under an administration supported by the rates. Again, they held that the Bill did not contain adequate provision for the treatment of those in sickness whom it desired to help. Immediately the insured person required hospital treatment the Bill threw him upon charity. Why should the Bill make provision for the treatment of consumption in sanatoria, which were acknowledged on all hands to be more or less experimental, and ignore the treatment of insured persons in hospitals or institutions, which were known to be absolutely necessary, and the efficacy of which was already proved? With regard to the expected depletion of hospital revenue, the speaker said that at the Western Infirmary, Glasgow, last year the total ordinary income, of which about 30 per cent. was contributed by employés, did not meet the total ordinary expenditure by 40 per cent., and the deficit was made up, 70 per cent. from legacies and 30 per cent. by a withdrawal from capital. The hospital had about 600 beds, and its ordinary expenditure was over £39,000 a year. He had less fear of the workmen withdrawing their subscriptions than he had of the employers, but he was most apprehensive of all about the legacies, for it was legacies which had made the existence of the hospital possible for thirty-seven years. They knew how little it took to divert interest from one object to another on the part of those leaving money, and if those about to make their wills got it into their heads that the days of the voluntary hospitals were numbered, they would not leave them money, and thus the crisis would be precipitated. It was questionable whether anything they could say or do would prevent the National Insurance Bill becoming law within a very short time. Whether their protest availed or not, they as hospital managers had a duty to the public to point out that the Bill as it stood was not adequate for the work it professed to do, and to protest as trustees of very large sums of money against a measure which, in their opinion, jeopardised the efficient carrying on of those institutions. If the good ship of the voluntary hospital, which had carried into safety and renewed health hundreds of thousands of those who had entrusted themselves to it, were to go down in the quicksands of the National Insurance Bill—and they knew what shifting sands they were—they would at least have the satisfaction of knowing that they had hoisted the signal of distress. (Applause.)

### MR. JAMES BOYTON, M.P., said that he had eleven hundred medical men in his constituency (East Marylebone), and spoke in support of the continuance of the voluntary hospital system. While sharing the ignorance of other speakers as to what this extraordinarily complex Bill might ultimately contain, he thought it was admitted that it dealt a blow at the voluntary hospitals all over the country. He also referred to the provision the Bill made for sanatoria, and suggested that, however worthy that particular work might be, there were surely other kinds of sickness and disease to the investigation and relief of which a special contribution might have been made.

Mr. W. G. Black (Glasgow Royal Infirmary and Glasgow Eye Infirmary) said that in Scotland in particular they were dependent upon the contributions of the working-men to a very large extent. The Glasgow Royal Infirmary had received over £24,000 in three years directly from the working-men of Glasgow. In the case of the Eye Infirmary half the revenue came from the same source. These were most momentous figures. The main purpose of the meeting was not only to protest but to consider what could be done. It was of no use discussing anything about the State hospitals. This was no part of the Chancellor's scheme. What they had to do was to consider as managers and directors of the hospitals up and down the country what policy they should pursue in face of the reduction in revenue. There was bound to be a reduction in the efficiency of the voluntary hospitals if the Bill were carried, and he thought that the member of Parliament who had neglected to safeguard the interests of the hospitals would very rightly meet with condemnation when, after a year's working of the Act, the position of his local infirmary was seriously weakened.

The Rev. H. V. Stuart (North Staffordshire Infirmary) moved an amendment which, he explained, was not in contravention of the spirit of the resolutions. He suggested the words, "are to be adequately provided for," instead of "are to be covered by the Bill," at the end of the first resolution. As it stood it suggested that the meeting put itself in favour of some contribution, and if they received payment for particular insured persons he did not see how they were possibly to escape control.

The amendment was seconded by Dr. T. B. Rhodes (North Staffordshire Infirmary).

Colonel T. W. Harding (Cambridge) said that he did not like the amendment much more than the original resolution. If they were in the presence of Mr. Lloyd George he would ask them, "What suggestions have you to make? What do you want?" And the speaker's suggestion would be that he withdraw the Bill and substitute a tentative Bill dealing with persons not earning more than thirty shillings a week.

The amendment was put to the meeting and lost by a large majority.

After the passing of the resolution some desultory discussion took place, one speaker, who expressed the opinion that the Chancellor of the Exchequer was not to be trusted as a negotiator and that "he was hoodwinking the doctors," appealed to the Association to "put some more fight into this business and to pass an even stronger resolution, suggesting that even hospitals would strike."

Sir Henry Burdett pointed out, in reply to criticisms, that it was the British Hospitals Association which had convened that meeting, and that everything which could be done was being done to put forward the hospitals' point of view. He reminded them that it was not only the voluntary hospitals throughout the country which would be damaged by the passing of the measure, but also the working classes, who would thus be deprived of the hospital care they now enjoy. He thought that every representative present should make the latter fact well known to the working classes throughout their districts. In this way the real effects of the Bill and the right impression of their attitude as hospital managers would become widely known.

Dr. T. B. Rhodes (North Staffordshire Infirmary) pointed out that by the resolution they had just passed the meeting committed itself to State aid. This being the case, it would be well to do something tangible side by side with a resolution, and he proposed that an Executive Committee be formed by the Council to follow up these arguments and press them upon the Government and the Chancellor of the Exchequer. The Rev. H. V. Stuart (North Staffordshire Infirmary) seconded. The resolution, however, was not put to the meeting, and it was understood that the matter was left for the Council to deal with.

The Rev. H. V. Stuart proposed that it be an instruction to the Executive Council that they should send these resolutions to the governors of the hospitals represented at that meeting in order that they should be as widely circulated as possible.

Sir Henry Burdett said that this had already been done, and, indeed, an extensive propaganda had been undertaken.

One speaker suggested that the friendly societies also should be circularised, but the honorary secretary pointed out that the British Hospitals Association, as a voluntary organisation, had no funds for such a purpose. The circularisation must be carried out as far as possible through individual efforts of each voluntary hospital and the Press.

Mr. Stuart withdrew his resolution, and the meeting broke up after a vote of thanks had been accorded to the chairman.

